# Incorporation of Personal Single Nucleotide Polymorphism (SNP) Data into a National Level Electronic Health Record for Disease Risk Assessment, Part 2: The Incorporation of SNP into the National Health Information System of Turkey

**DOI:** 10.2196/medinform.3555

**Published:** 2014-08-11

**Authors:** Timur Beyan, Yeşim Aydın Son

**Affiliations:** ^1^Informatics InstituteDepartment of Health InformaticsMiddle East Technical UniversityAnkaraTurkey

**Keywords:** health information systems, clinical decision support systems, disease risk model, electronic health record, epigenetics, personalized medicine, single nucleotide polymorphism

## Abstract

**Background:**

A personalized medicine approach provides opportunities for predictive and preventive medicine. Using genomic, clinical, environmental, and behavioral data, the tracking and management of individual wellness is possible. A prolific way to carry this personalized approach into routine practices can be accomplished by integrating clinical interpretations of genomic variations into electronic medical record (EMR)s/electronic health record (EHR)s systems. Today, various central EHR infrastructures have been constituted in many countries of the world, including Turkey.

**Objective:**

As an initial attempt to develop a sophisticated infrastructure, we have concentrated on incorporating the personal single nucleotide polymorphism (SNP) data into the National Health Information System of Turkey (NHIS-T) for disease risk assessment, and evaluated the performance of various predictive models for prostate cancer cases. We present our work as a miniseries containing three parts: (1) an overview of requirements, (2) the incorporation of SNP into the NHIS-T, and (3) an evaluation of SNP data incorporated into the NHIS-T for prostate cancer.

**Methods:**

For the second article of this miniseries, we have analyzed the existing NHIS-T and proposed the possible extensional architectures. In light of the literature survey and characteristics of NHIS-T, we have proposed and argued opportunities and obstacles for a SNP incorporated NHIS-T. A prototype with complementary capabilities (knowledge base and end-user applications) for these architectures has been designed and developed.

**Results:**

In the proposed architectures, the clinically relevant personal SNP (CR-SNP) and clinicogenomic associations are shared between central repositories and end-users via the NHIS-T infrastructure. To produce these files, we need to develop a national level clinicogenomic knowledge base. Regarding clinicogenomic decision support, we planned to complete interpretation of these associations on the end-user applications. This approach gives us the flexibility to add/update envirobehavioral parameters and family health history that will be monitored or collected by end users.

**Conclusions:**

Our results emphasized that even though the existing NHIS-T messaging infrastructure supports the integration of SNP data and clinicogenomic association, it is critical to develop a national level, accredited knowledge base and better end-user systems for the interpretation of genomic, clinical, and envirobehavioral parameters.

## Introduction

In clinical decision processes, genomic variant data can be used for assessing disease risks, predicting susceptibility, providing targeted screening and early diagnosis, and for planning treatment regimens [1,2]. A reasonable way to implement this personalized approach into a routine for medical practices would be to integrate genotype data and its clinical interpretation into the electronic medical record (EMR)/electronic health record (EHR) systems [3,4].

Today, in many developed and developing countries, the use of EMRs/EHRs is essential for health care providers for reimbursement of services and to track the quality of the health care provided [5,6]. Recently, several EHR networks have been established in many countries of the world, including the National Health Information System of Turkey (NHIS-T) [7]. These EHR systems and networks have high potential for integrating genomic data in health care practices for personalized medicine.

The aim of this miniseries is to present how the incorporation of personal single nucleotide polymorphism (SNP) data into the NHIS-T would make disease risk assessment possible, and to evaluate the performance of various predictive models for a specific medical condition (eg, prostate cancer). The requirements of SNP data integrated with EMRs/EHRs from scientific literature have been reviewed in the previous part of this miniseries [8], and here we will focus on extending the capabilities of the NHIS-T via incorporating SNP and clinicogenomic data for disease risk assessment. In the final part of the miniseries, we will evaluate the proposed complementary capabilities with real data from prostate cancer cases [9].

## Methods

In this part of the miniseries, we studied existing NHIS-T regarding architecture, standards, and terminologies. We explained data elements, minimum health datasets, transmission sets, and the National Health Data Dictionary and their roles to produce a conceptual EHR design. Then, we clarified the messaging infrastructure and serialization approach of NHIS-T based on Health Level 7 (HL7) Clinical Document Architecture (CDA) standard.

After that, we argued the possible architectural extensions with complementary capabilities for a SNP data incorporated NHIS-T in the light of literature review and characteristics of NHIS-T [8].

After the presentation of a general use case for our approach, we put forward the design and development efforts for the complementary components, namely, knowledge base (Clinicogenomic Knowledge Base, ClinGenKB) and end-user application (Clinicogenomic Web Application, ClinGenWeb). In this phase, we have constituted the standardized definition tables for clinicogenomic associations and predictive models for these complementary capabilities.

Through analysis of the disease risk approaches from literature for prostate cancer, we have extracted possible clinicogenomic association types for assessment and reporting. At the end, we have generated a comparative table to determine the requirements to produce a standard representation for all types of clinicogenomic associations.

In addition, to interpret clinicogenomic associations at the end-user side (ClinGenWeb) using various predictive models, we designed a standardized model definition table. In both definition tables, we determined terminology standards for data elements.

In the final phase, to develop the ClinGenKB we used BioXM Knowledge Management Environment (BioXM), which is a distributed software platform providing a central inventory of information and knowledge [10]. Also, we have developed a practical reporting approach and demonstrated it using Zoho Reports as a prototype system (namely, ClinGenWeb) for the client side [11].

## Results

### Analysis of National Health Information System of Turkey

#### Overview

NHIS-T is a national level health information infrastructure that has a centralized service-oriented architecture in order to produce and share medical records among stakeholders [12,13].

Every care provider organization in Turkey has to collect patient/medical records in its EMR systems, and send some predefined structured medical data to the central Republic of Turkey’s Ministry of Health (MoH) databases. The architecture of local EMR systems varies because of the existence of different vendors in the market, and generally most of the clinical data is collected as narrative texts. But, because it is mandatory to conform to the NHIS-T standards while sending predefined datasets to the MoH servers, these are stored as structured data in the local EMRs.

In the NHIS-T, two standards are important: (1) the United Nations Centre for Trade Facilitation and Electronic Business (UN/CEFACT) Core Component Technical Specification (CCTS) to design EHR content in the conceptual base, and (2) HL7 CDA to serialize this conceptual design.

#### Design of the Electronic Health Record Content

UN/CEFACT CCTS is a methodology to define the structured, abstract document components used to increase the interoperability of electronic business documents. In the NHIS-T, EHR content is designed based on UN/CEFACT CCTS to assure the reuse of common information blocks in EHRs. First, the data types and the data elements used in EHRs are identified, and then a set of reusable building blocks of the EHRs, named the Minimum Health Datasets (MHDS), is produced. Some examples of the MHDS are maternal mortality dataset, diabetes dataset, dialysis patient dataset, patient admission dataset, cancer dataset, chronic disease dataset, etc.

In every dataset, there are many data elements. For example, data elements of cancer datasets are data of first diagnosis; diagnostic method; location histological type; the Surveillance, Epidemiology, and End Results Program (SEER) summary stage; laterality; occupation; and cancer.

The data elements are coded with universal medical terminologies (eg, International Classification of Diseases and Health Related Problems version 10, ICD-10; Anatomical Therapeutic Chemical, ATC; etc) and predefined categorical values standardized by the MoH, such as gender or marital status. All kinds of these terminologies are selected by the MoH and available from the Health Coding Reference Server (HCRS). There are 342 code systems in the HCRS; the current version of the HCRS is 3.0 and is available on the Internet via Web services. A tabular representation is also available on an official Web page and allows users to query by means of Web browsers.

These reusable building blocks (MHDS) are assembled into aggregate document components called Transmission Datasets (TDS, episodic EHRs). Some of the TDS are physical examination TDS, laboratory test results TDS, and in-patient TDS.

All the data elements, MHDS, and TDS are identified by the MoH in the light of the needs of stakeholders (eg, strategic decision makers, health care organizations, academic institutions, etc) and published in the National Health Data Dictionary (NHDD). This is a dynamic and continuously improving process. When required, new MHDS are produced using existing data elements, and the NHDD is improved by identifying new data elements. The total number of data elements, MHDS, and TDS in the most recent version of NHDD; namely, version 2.2, are 418, 66, and 7, respectively. All versions of NHDD are available on the official website of The Turkey e-Health project [14].

#### Transport of the Electronic Health Record Content

This conceptual EHR architecture is serialized into extensible markup language (XML) based on the HL7 CDA structure. As described in the first part of this miniseries, HL7 CDA is a document mark-up standard referred to in the exchange of information as part of the HL7 version 3 (V3) standards that aim to specify the structural and semantic aspects of clinical documents [15].

In the serialization process, the TDS are mapped to HL7 CDA to create the “transmission schema”. Each transmission schema is wrapped with a root element named after the main dataset in the transmission [12,13].

The NHIS-T messaging system accepts HL7 V3 CDA release 2 (R2) as a reference and is compliant to this standard. Therefore, the messages sent must comply with the rules defined in the CDA XML Schema Definition file. Health care organizations send messages containing clinical data to the central MoH servers through Web services. In the current version of the NHIS-T system, the transmission schema instances are localized according to Turkey’s HL7 Profile. During this process, the rules, which are set in the “HL7 Refinement, Constraint, and Localization” document, are applied. All of these templates are created and published by the MoH, and used as the standard by health care information systems vendors [12,16].

**Figure 1:**
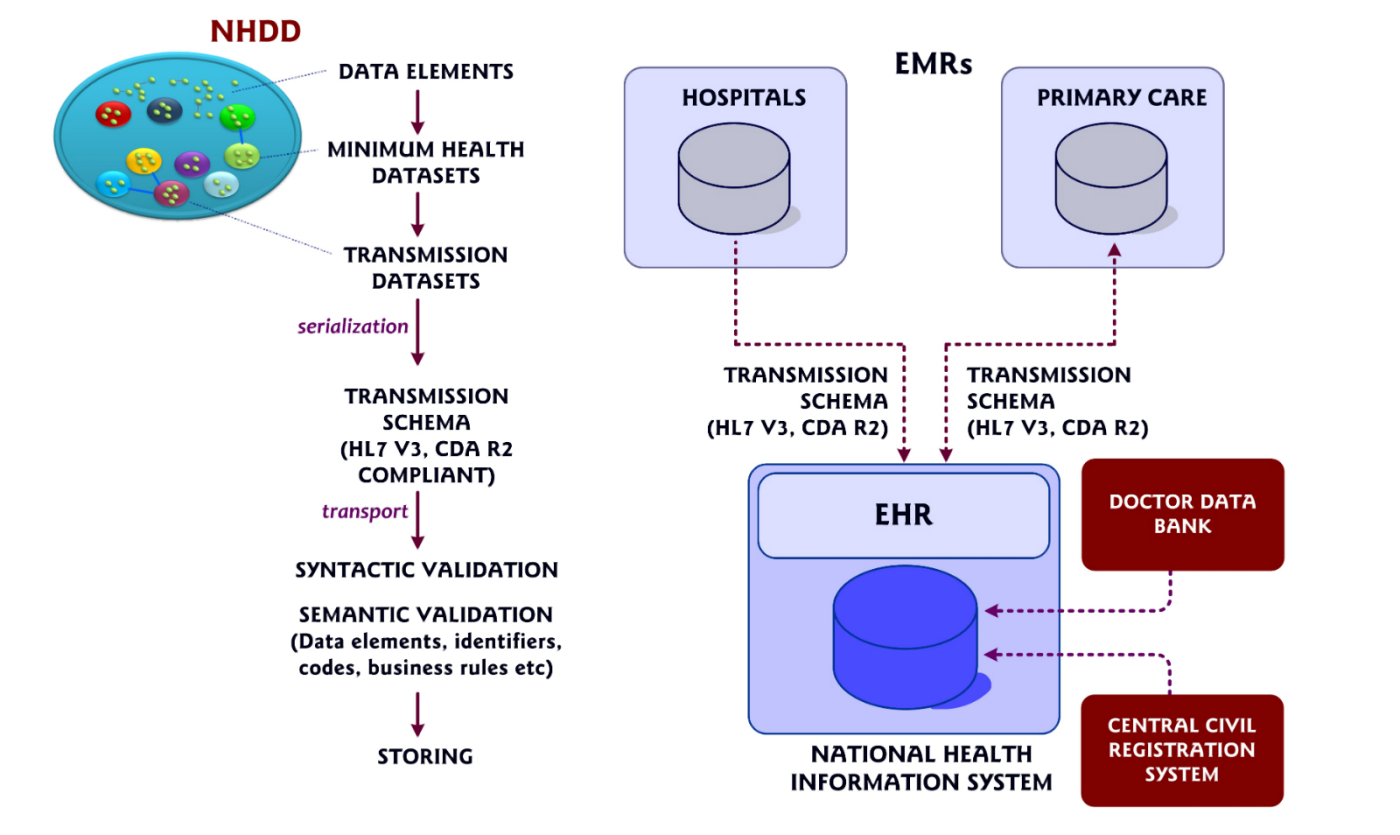
Schematic representation of National Health Information System of Turkey. NHDD=National Health Data Dictionary; HL7 CDA R2=Health Level 7 Clinical Document Architecture release 2; EMR=electronic medical record; and EHR=electronic health record.

Incoming messages are validated regarding syntax and semantics, and the appropriate messages are stored in the NHIS-T central repositories. Patient and medical professional identifiers are acquired and validated from the Central Civil Registration System and Doctor Data Bank, respectively (Figure 1 shows the NHIS-T) [13,16].

The current version of the NHIS-T messaging system allows for the transfer of medical data from health care providers’ (hospitals and family practitioners) information systems to central servers via Web services. It has the infrastructure that will provide access to patients’ records for authorized health care professionals within the hospital, which will allow patients to reach their own medical data, for example, personal health records (PHRs). But, the legal regulations have to be completed before both types of access—authorized or self—are available. Then, the establishment of a PHR system will be allowed [13].

### Architectural Extensions for Single Nucleotide Polymorphism Data Incorporated National Health Information System of Turkey

It is necessary to build an infrastructure providing clinicogenomic information and subsequent updates to the physicians. A curated knowledge base extracting clinical information from relevant SNP data, and supporting sys­tems processing up-to-date data for clinical decisions over the patient’s lifetime should be integrated as clinicogenomic information into medical records [17,18].

In the light of our literature review, for a genome-enabled NHIS-T, improvements in three components are needed: (1) enhancement of existing messaging infrastructure to share personal SNP data and clinicogenomic associations between stakeholders, (2) development of a national-level ClinGenKB for transforming personal SNP data to clinicogenomic associations, and (3) advancement of end-user applications (EMR, PHR, etc) for reporting of clinicogenomic interpretation of clinically relevant SNP data.

A messaging infrastructure ensures the connection of different stakeholders and the sharing of all types of relevant data among these partners, for example, relevant SNP data between genomic laboratory and knowledge base, and clinicogenomic associations between knowledge base and end-user applications.

As clinicians cannot extract the clinical interpretation of all SNP variations directly from the medical sources due to temporal and cognitive limitations, the integration of the clinical interpretations of variations (eg, clinicogenomic associations) into the medical record will be more efficient for clinical decision making [19-22]. To convert SNP data into clinicogenomic associations and infer clinically meaningful results, we need a structured knowledge source, for example, a knowledge base.

Most of the clinically relevant SNPs have minor effects (odds ratio <1.50-2.00), and there are only limited numbers of different examples. For example, in our study, we extracted more than 209 prostate cancer-associated SNPs from the literature, and many of these SNPs have minor effects for predicting the prostate-cancer risk [9]. Additionally, in many cases, SNPs do not show their effects directly for a given disease, but do so in combination with other SNP variations and clinical and environmental factors, which require a much higher level of probability calculations.

Previously, various approaches were proposed to assess and report clinicogenomic associations. The listing of clinicogenomic associations and their effects may be useful for a limited number of independent associations. But, it is clear that clinicians cannot interpret and evaluate all variations individually, especially for SNPs with small impact degrees.

Each day, the volume of variation data integrated into clinical practice exceeds the boundaries of unsupported human cognition and interpretive capacity. Additionally, the rapidly growing literature on clinicogenomic associations increases the challenge for professionals to stay current.

Therefore, the polygenic risk models that are under development or panels, which assign values for various SNP alleles and calculate the total risk of diseases, will be more effective for risk prediction. Eventually, we also need end-user applications that report clinicogenomic associations independently and risk-value calculations based on predictive models.

Regarding technical capabilities (eg, network bandwidth, storing and processing capacities, etc), different types of architectures can be developed, but the development of two additional components (knowledge base and reporting capability) is essential. A ClinGenKB must be constructed at the national level as a manually curated and continuously updated source that would include clinical information and its possible associations with SNP variants. In the end-user, decision-support applications (EMRs, PHRs, etc), clinicogenomic associations, and external data (eg, family history) must be interpreted independently, or based on predictive models to support decision making.

In the existing NHIS-T, medical and laboratory test results are sent from hospitals to the central EHR databases as “Examination Result Transmission Dataset”. The HL7 CDA R2 conformant transmission schema of this dataset includes several MHDS, for example, registration MHDS, result of tests MHDS, patient MHDS, etc. “Result of tests MHDS” involves data elements about examination features (order time, protocol number, result time, test result, reference value ranges, etc). The data type of laboratory analysis should be numeric or textual data regarding current schema standards. HL7 V3 interoperability standards support encapsulated data type for text data [15].

**Figure 2:**
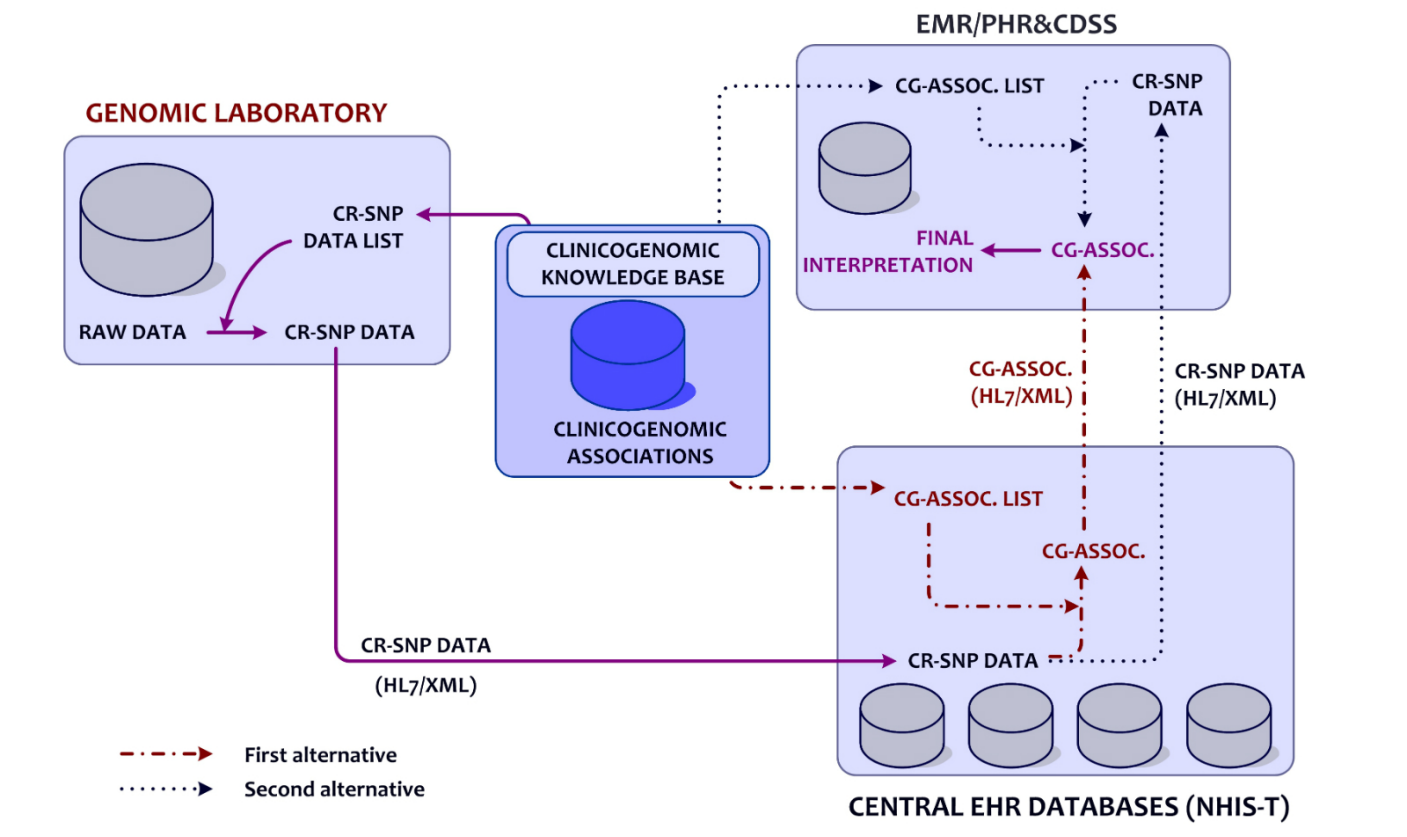
Alternatives for extended architecture of genome enabled National Health Information System of Turkey (NHIS-T). CR-SNP=clinically relevant single nucleotide polymorphism; HL7=Health Level 7; XML=extensible markup language; CG-ASSOC.=clinicogenomic associations; EMR=electronic medical record; PHR=personal health record; and CDSS=clinical decision support system.

Although whole genome sequencing (WGS), whole exome sequencing (WES), and other types of genotyping tests are accepted as laboratory tests, they have different characteristics than other laboratory tests in routine practice. After a clinical WGS/WES test, a personal SNP data file which contains a large amount of variant data is produced, in which all variant data needs to be managed in an effective way. In the proposed architecture, personal sequencing data is acquired and stored as raw data within a genomic laboratory information system. The clinically relevant personal SNP (CR-SNP) data is extracted from a personal SNP data file using the CR-SNP data list (genomic identifiers of clinicogenomic associations in the ClinGenKB). Then, a personal CR-SNP data file is sent via the NHIS-T infrastructure from a genomic laboratory to central EHR databases as an encapsulated text file. This file has to include SNP identifiers, for example, reference SNP identifier (rsID) and allele data in the HL7 CDA R2 schema (Figure 2 shows this process).

The received CR-SNP files would be stored within the central EHR databases. Then, the CR-SNP files can be processed to infer clinically relevant data by using the clinicogenomic associations from the knowledge base. Resulting personal, clinicogenomic association files would be sent to end users. As shown in Figure 2, based on existing technical capabilities, to decrease the load of sharing clinicogenomic association files, a replicated knowledge base could be integrated as a Web application running on the client side, whereas a CR-SNP data file could only be stored in central servers. In this situation, clinicogenomic associations are inferred at the client side through the replicated knowledge base. In that case, the client-side knowledge base must be frequently synchronized with the central knowledge base.

To link personal CR-SNP data and clinicogenomic associations, the rsID and allele combination is used. Data input to the knowledge base rules (or associations) require rsID and allele information, and medical interpretation, significance, and representative information are sent back as the output from the knowledge base. The rule structure is explained in the section on “Definition of Clinicogenomic Associations”. Finally, personal clinicogenomic associations are inferred from personal CR-SNP data and a knowledge base.

When an authorized user (patient, family practitioner, or a medical specialist) needs to reach a personal CR-SNP or a clinicogenomic association file, a request should be sent to the central EHR, and a current data file would be received via the NHIS-T communication infrastructure.

Additionally, it is recommended that an independent medical authority, established by domain experts, should update ClinGenKB. According to the type and level of change and preferred architecture, existing personal SNP data, CR-SNP data, and/or clinicogenomic associations must be reinterpreted after the authorization of the patient, through the genomic laboratory system at the national EHR repository and/or the client side.

Our assessment of the NHIS-T reveals that its capabilities (eg, regarding Web services, client-side inference, and reporting capabilities, PHRs, if requested) need to be extended to be able to share CR-SNP or personal clinicogenomic associations between central EHR databases and end-user systems. Here, we are presenting complementary capabilities developed as prototypes for the NHIS-T, for example, ClinGenKB and ClinGenWeb, which specifically focus on disease risk assessment. In our study, as an initial attempt through the development of much sophisticated infrastructure, we have concentrated on the interpretation of SNP variant data and excluded other types of variants. The use of personal clinicogenomic information to determine the disease risk of a patient’s family members is considered to be out of scope. Also, security and privacy issues, as well as constraints about hardware and infrastructure, are excluded.

### Design of the Complementary Components

#### General Use Case

**Figure 3:**
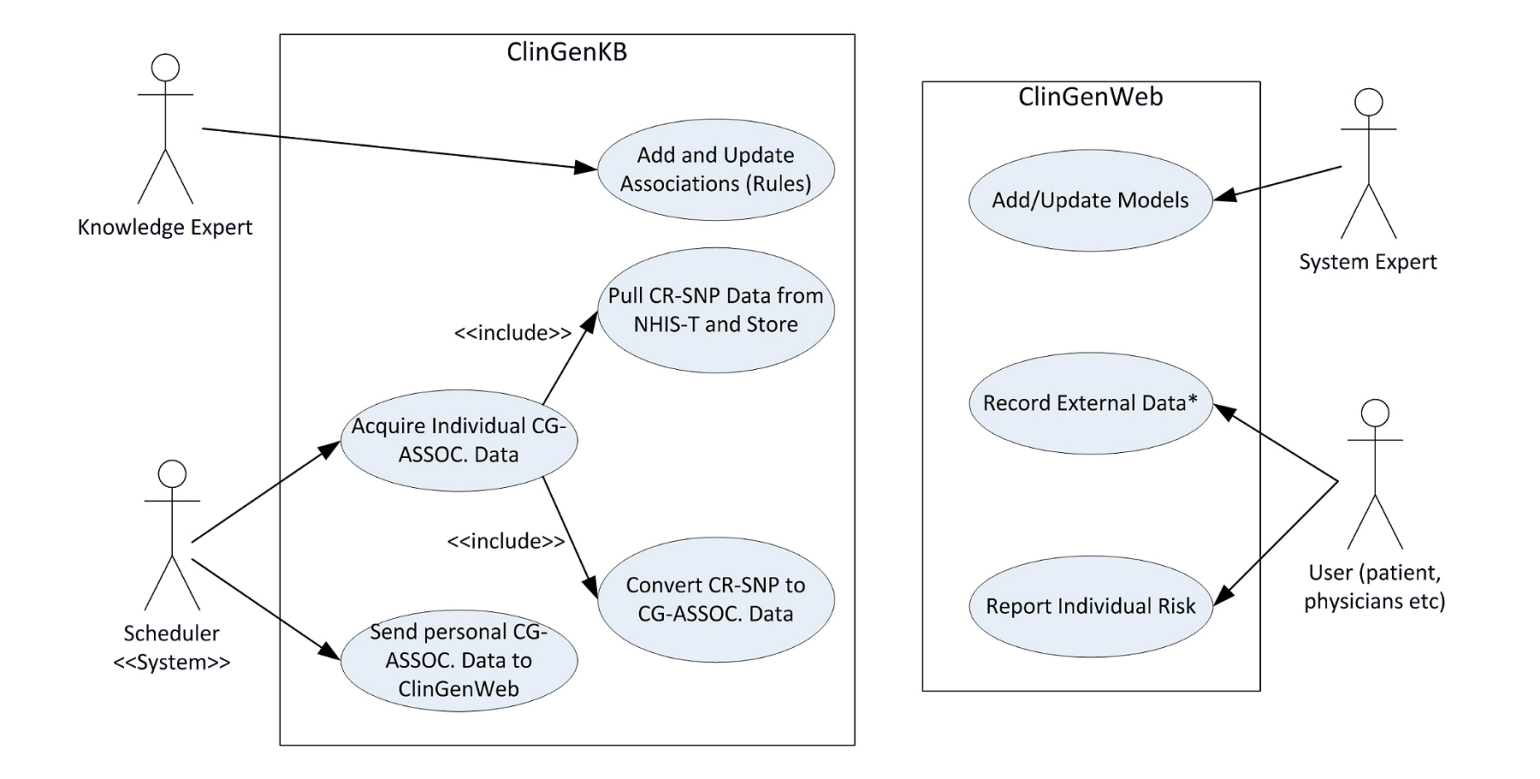
The use case diagram of our Clinicogenomic Knowledge Base (ClinGenKB) and Clinicogenomic Web Application (ClinGenWeb). CR-SNP=clinically relevant single nucleotide polymorphism; CG-ASSOC.=clinicogenomic associations; NHIS-T=National Health Information System of Turkey; and *External Data: environmental, behavioral, family health history data, etc.

Figure 3 shows the general use case diagram of our approach. Major actors of our system are the end-users (physicians or patients), knowledge expert, and system expert. The NHIS-T infrastructure, ClinGenKB, and ClinGenWeb will perform the sending and storing functionalities. Knowledge experts add and update clinicogenomic associations into the ClinGenKB. The conversion process of the CR-SNP to the clinicogenomic associations is accomplished synchronously by ClinGenKB at the first uploading and then—as a rule—in update sessions.

Before the design and development of the ClinGenKB and the ClinGenWeb, we standardized the clinicogenomic associations and models, as explained below.

#### Standardization of the Clinicogenomic Approaches

At the end-user side, assessment and reporting of clinicogenomic associations using several approaches, for example, listing of the independent associations, complete visualization of independent associations, polygenic risk scoring, and model-based methods, etc, were determined as requirements of the system. The common data fields and approach-specific additional data fields of clinicogenomic associations in the ClinGenKB must be defined for handling a variety of approaches. In particular, the ClinGenKB should collect all types of independent and model-based clinicogenomic associations as a whole set. So, in our study, we reduced the independent and different types of model-based associations into a standard definition while designing the ClinGenKB.

In the scientific literature, we have determined two types of models, for example, cumulative and probabilistic models, for prostate cancer risk assessment. In cumulative models, we can calculate the possible disease risk by combining the impact of several clinicogenomic associations. In a probabilistic model, SNP profiles with increased disease risk are determined with an evidence-based approach during development of the model, and the patient’s risk is determined through the patient’s genotyping profiles. Detailed examples of these models will be explained in the next section of the miniseries [9]. Examples of the cumulative model of prostate cancer and their reference tables are given in Multimedia Appendices 1 and 2, and examples of the probabilistic model with the list of the possible SNP profiles are provided in Multimedia Appendices 3 and 4.

Through analysis of these models, we have extracted possible clinicogenomic association types for assessment and reporting. Finally, we have generated a comparative table to determine the requirements for the design of the ClinGenKB (Table 1).

**Table 1:** Comparison of representation types and definitive data fields.

Definitions		Independent associations	Complete visualization for independent associations	Associations of cumulative models	Associations of probabilistic models
**Association**					
	rsID	X	X	X	X
	Allele	X	X	X	X
	Disease code	X	X	X	X
	Disease name	X	X	X	X
	Magnitude of impact	X			
	Degree of evidence quality	X			
	Impact category		X		
	Evidence category		X		
	Impact value				
	Branch_id^c^				X
**Model**					
	Model type	X	X	X	X
	Model name	X^a^	X^a^	X	X
	Total impact			X	
	Total count of SNPs				X
	Branch_id				X
	Narrative interpretation			X	X
**External data**					
	Family history			X	X
	Other type^b^				X

^a^In this study, only increased risk covered

^b^BMI = body mass index, alcohol consumption, smoking, etc

^c^Branch_id, a numeric identifier for every association which is derived from a probabilistic model.

#### Definition of the Clinicogenomic Association

Next, we have produced a standard representation for all types of clinicogenomic associations, including an association identifier, genomic parts, and clinical parts. Genomic parts contain SNP data, namely, rsID and allele values. In the clinical part, there are medical data, model information, values about impact, and evidence degree of per association.

Detailed data analysis of association typology is presented in Table 2. This table was used while designing the ClinGenKB.

**Table 2:** Data field analysis of association parameters.

Data category	Parameters	Values
Association identifier	Assoc_id	Unique numeric value
SNP data	rsID	rs value
	Allele	Allele value (eg, AA^e^; AT^f^; , CG^g^; etc)
		
Medical data	Disease code and name	ICD-10
		
Representation model of associations	Model type	Independent associations, cumulative model, and probabilistic model
	Model name	Increased^a^ ; (number of SNP and name of first author)^b^; (model number, name of authors, and date)^c^
Association values	Parameter 1 (magnitude of impact^a^; impact value^b^; and branch_id^c^)	Numeric value^a, b^; numeric identifier^c^
	Parameter 2 (degree of quality of evidence^a^)	Numeric value (between 1 and 3)
	Parameter 3 (impact category^d^)	1,2,3 (corresponding Weak, Moderate, Strong, respectively)^d^
	Parameter 4 (evidence category^d^)	1,2,3 (corresponding Weak, Moderate, Strong, respectively)^d^

^a^Independent associations

^b^Cumulative model-based associations

^c^Probabilistic model-based associations

^d^Complete assessment for independent associations

^e^AA = adenine-adenine

^f^AT = adenine-timine

^g^CG = cytosine-guanine

#### Data Fields of a Clinicogenomic Association

In this representation, clinicogenomic associations must have a unique identifier assigned automatically. In ClinGenKB, SNPs are identified by both rsID and allele data that correspond to the forward strand of genomic sequence. If a SNP is related with the different medical conditions or models, for every instance, a new association was defined, and a different unique identifier was assigned.

The medical data category contains diagnosis codes and names. Values from this data field are selected from the ICD-10, which is used for diagnostic terminology for diseases in the current NHIS-T.

Model data has two components: (1) type, and (2) name. In ClinGenKB, we have two main clinicogenomic associations, for example, model-based or model-free (or independent) associations. Names for independent associations are categorized as increased or decreased regarding the potential risks and/or protective characteristics. In this study, we have focused only on increased risk. Model-based associations are used in the predictive models.

Association values are tightly related to the type of model, for example, independent associations, cumulative model, and probabilistic model-based associations. For an independent associations odds ratio (OR), the degree of evidence quality, impact values, and evidence categories were found to be appropriate and sufficient elements to evaluate clinical significance, both individually and as a whole. In cumulative model-based associations, it is necessary to assign an impact value for every association to calculate the total personal risk value according to the model-definition table. For the probabilistic model, we have calculated the total effects of variants using their branch_id. On the client side, all possible associations derived from the probabilistic model are grouped by “branch_id”, and for all of these groups, the total impact of risk parameters is determined. If one of these values is equal to the total value of the corresponding branch, it is interpreted as the patient having the risk of prostate cancer, based on the accuracy, precision, and recall values of the model. For example, in the only SNP model, 154 different possibilities were defined. According to the first branch (branch_id=1), if an individual carries all of the “rs11720239-AA, rs2999081-CT, rs2811518-CT, and rs4793790-TT” SNP variations, it is assessed as this individual having a risk of developing prostate cancer with the degree of the accuracy, precision, and recall of the model.

#### Predictive Risk Models

Additionally, a standardized model definition table involving reference values for variants and corresponding disease risks is produced, as the final interpretation of clinicogenomic associations will be completed at the end-user side (ClinGenWeb) using predictive models.

We have developed the model definition table for the analysis of the model-based associations on the client side (Table 3). The model type identifies the category of models, and model name labels them. The total value and explanation fields are mapped to the total impact of related SNPs and the corresponding risk categories. For cumulative models, these fields are about total impact and its explanation. For the probabilistic models, total value is referred to as the count of all SNPs for every branch; the explanation is the interpretation of risk values regarding accuracy and precision. An additional data field identifying the branch_id of the selected support vector machine- iterative dichotomiser 3 hybrid model is needed for the explanation of the probabilistic model.

Examples of cumulative models are given in Multimedia Appendices 1 and 2, and a list of examples of probabilistic models and their parameters is given in Multimedia Appendices 3 and 4.

**Table 3:** Data field analysis of model definition table. OR: odds ratio.

Parameters	Value (domain)	Explanation
Model type	Cumulative model, probabilistic model	
Model name	(Number of SNP and name of first author)^a^; (model number, name of author, and date)^b^	
Total value	Numeric value	Total impact^a^; total count of SNPs^b^
Explanation 1	Text value	Explanation (OR)^a^; Explanation (brief interpretation about risk assessment)^b^
Explanation 2	Text value	Explanation (branch_id)^b^

^a^Cumulative model-based associations

^b^Probabilistic model-based associations

### Development of the Complementary Components

#### Clinicogenomic Knowledge Base

Knowledge bases are repositories that help to collect, organize, share, search, and utilize information. Developing an accurate, accessible, structured clinicogenomic knowledge source (ClinGenKB) for disease-associated SNPs (prostate cancer in our case) is an essential component of the proposed clinicogenomic information-integrated EHR.

Raw genomic variant data is not appropriate to support clinician decision-making due to its high dimension. The clinical association of the variant is convenient to transfer for clinical decision support, where the interpretation of the variant and its associated clinical meaning is periodically updated in the knowledge base. The data field tables described in Figure 3 must be kept up to date and shared with other stakeholders to be used as a standard reference for the interpretation of the model-based associations in a proper manner. Also, model type and model name fields must be used as standard references for the same fields in the definition tables. Such a system would allow for the reinterpretation of variant data throughout dynamic updates.

Technically, there are many tools for knowledge modeling and implementation. For this study, we have preferred to develop our prototype using BioXM, which is a distributed software platform providing a central inventory of information and knowledge [10]. Through BioXM, we were able to quickly generate, easily manage, and visualize the scientific models as extendible networks of interrelated concepts.

To develop the ClinGenKB on the BioXM platform, we have designed the domain-specific data model with semantic objects—elements, annotations, ontologies, and databanks—and the connections (relations) using the BioXM graph viewer based on our clinicogenomic association definitions. Next, we have defined the importing scripts to transfer the extracted independent and model-based clinicogenomic associations and the personal CR-SNP data to the knowledge base. BioXM supports the data import and export as XML, hyper text markup language, Excel, or plain text format. Finally, we have prepared views, queries, and smart folders to manage our data model and the inferring processes.

**Figure 4:**
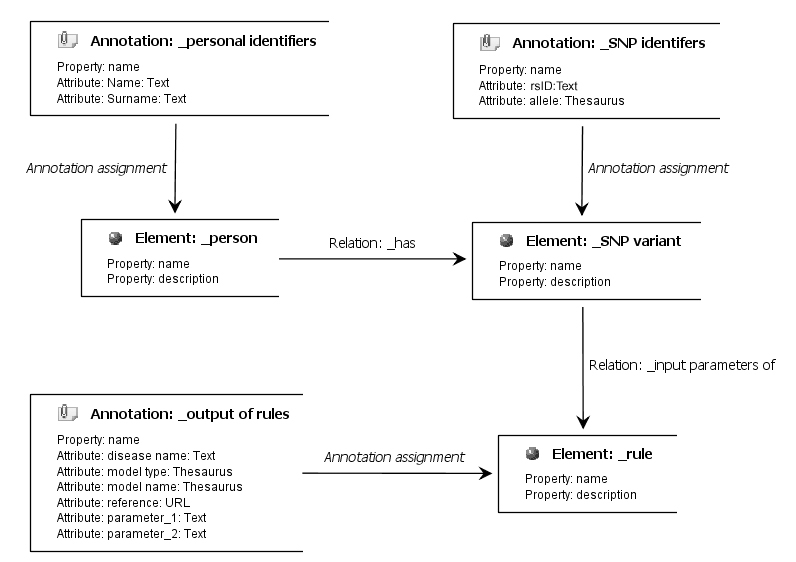
The graphical representation of designed prototype with BioXM Knowledge Management Environment. URL=uniform resource locator; rsID=reference single nucleotide polymorphism identifier; and SNP=single nucleotide polymorphism.

Our domain model defining elements, annotations, relations, and scope of these components in BioXM is based on the association definition table (Figure 4 shows this table). In this domain model, we have three types of elements: (1) person, (2) SNP variant, and (3) clinical association. Every element has its specific annotations. The personal element is related with the SNP variant by a “has” relation, referring to the fact that each patient will have a set of SNP variants. SNP identifiers are assigned to variants for ensuring uniqueness. Then, each SNP variant is related with a clinical association element as the input.

**Figure 5:**
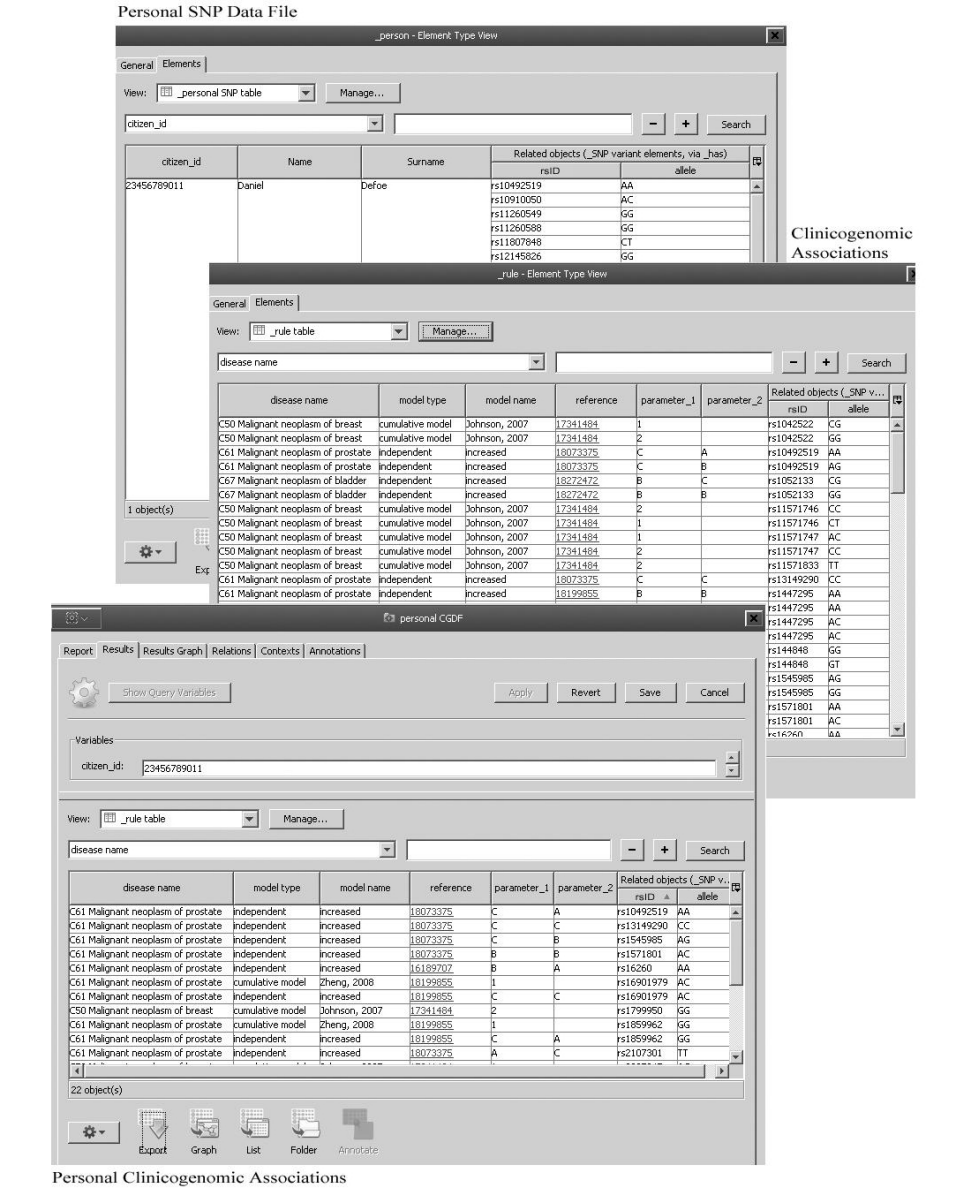
Functional steps for Clinicogenomic Knowledge Base (ClinGenKB): (1) defining clinicogenomic content, (2) uploading personal clinically relevant single nucleotide polymorphism (SNP) data, and (3) inferring personal clinicogenomic associations based on the content. rsID=reference single nucleotide polymorphism identifier; AA=adenine-adenine; AC=adenine-cytosine; AG=adenine-guanine; CC=cytosine-cytosine; CG=cytosine-guanine; CT=cytosine-thymine; and GG=guanine-guanine.

We have imported the content of the knowledge-based definition table as an external file with scripts. This content can be updated with subsequent importing operations. If a new association is generated or existing associations are changed or cancelled, authorities can organize all the changes in an external source according to the association definition table, and then can easily upload all of them via BioXM compatible files. After the importing process, the clinicogenomic associations can be sorted and managed by system administrator from table (Figure 5 shows a screenshot).

In addition, we can store personal CR-SNP data as a separate file on BioXM. CR-SNP data can be easily converted to clinicogenomic associations based on the content of ClinGenKB, and this data file is exported as a text file. For all individuals whose CR-SNP data is stored in BioXM, whenever it is needed, it is possible to access personal CR-SNP data, and to produce new clinicogenomic association data files based on the current ClinGenKB. In the NHIS-T, all of these personal files can be accessible with the inclusion of the Turkish citizen-identifier number in our prototype, and can be sorted according to data categories (Figure 5).

#### Clinicogenomic Web Application

After the transmission of the personal, clinicogenomic association data file to the end-users’ (medical specialists, family practitioners, and patients) applications, another critical issue is the final interpretation and reporting of the results. Reporting presents itself here as a critical point for maximizing the effectiveness of the overall system in translating clinicogenomic data into the clinic. High-dimensional variant data and its clinical associations—along with its interpretations—have to be reported and visualized in a simplistic and holistic manner for easy interpretation by both health care professionals and patients.

Regarding clinicogenomic decision support, our approach aims to divide clinicogenomic interpretation into two phases, namely: (1) conversion of the variant SNP into a clinicogenomic association, and (2) clinical interpretation of these associations. Final interpretation is completed on the client side. This approach gives us the flexibility to add/update external parameters that will be monitored or collected by end users. For example, in some cumulative prostate models, positive family history augments the total risk value in addition to clinically relevant SNPs. Family history is a dynamic parameter that can change in time. Patients ideally accomplish effective tracking of changes in family history. Similarly, clinical, environmental, behavioral, or sociodemographic factors, should be involved to assess the total risk with variant data at the end-user level.

Accordingly, we have developed a practical reporting approach and demonstrated it using Zoho Reports as a prototype system (namely, ClinGenWeb) for the client side. Zoho Reports is an on-demand reporting and business intelligence tool that supports several, report generation capabilities, for example, chart/graph, tabular views, summary views, pivot tables, dashboards, and structured query language (SQL)-driven querying. Most importantly, it is possible to embed generated reports within external Web sites and Web applications [11]. ClinGenWeb is developed as a Web application, processing genomic associations and clinical and environmental risk parameters. ClinGenWeb is designed with the capability to report relevant clinicogenomic SNPs or to assess individual risk based on different models with the combination of conventional health data and clinicogenomic associations.

The ClinGenWeb is designed to report personal predictive risk under three main categories: (1) detailed reporting of individual associations, (2) the assessment of a number of clinically relevant SNPs, and (3) model-based interpretations of the clinicogenomic associations. Basic models are based only on assessing the relevant SNPs, whereas other predictive models require additional clinical data (family history, BMI, etc). When provided, corresponding risk factors for prostate cancer can be used to calculate the model-based risk. Also, external personal data about clinical and some environmental risk factors for prostate cancer can be reported.

#### Reporting of Independent Associations

**Figure 6:**
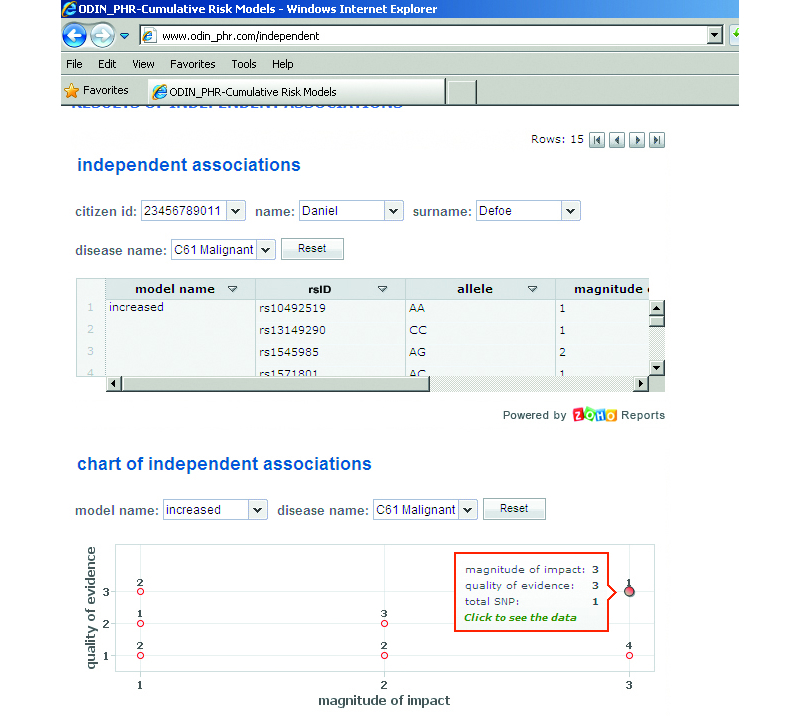
Visualization of independent associations in the Clinicogenomic Web Application (ClinGenWeb). Independent associations and their clinical significances are listed as a table and represented on a category based graph. rsID=reference single nucleotide polymorphism identifier; SNP=single nucleotide polymorphism; AA=adenine-adenine; CC=cytosine-cytosine; and AG=adenine-guanine.

The reporting of all independent associations individually would be very confusing, and the interpretation of this data by users would be time consuming. So, the associated data are presented in a category-based graph, where the x- and y-axes correspond to impact and evidence categories, respectively (Figure 6 shows this graph).

#### Reporting of Model-Based Associations

Comparatively, a model-based interpretation provides us with more effective information for supporting the end-users’ decision making. This type of rule is based on accepted and proven integrated models as described in the next section.

**Figure 7:**
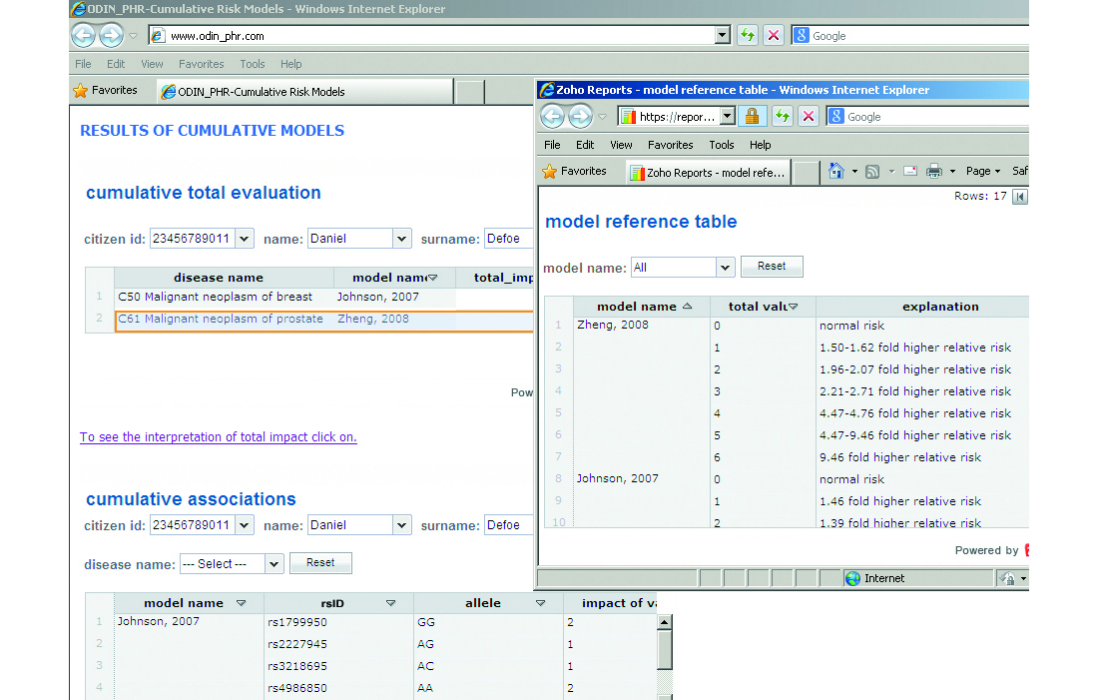
Reporting and interpretation of the results of model-based associations and the whole impact (and meaning) of models in the Clinicogenomic Web Application (ClinGenWeb). rsID=reference single nucleotide polymorphism identifier; AA=adenine-adenine; AC=adenine-cytosine; AG=adenine-guanine; and GG=guanine-guanine.

In our study, we have used two kinds of models, namely, cumulative and probabilistic models. In the ClinGenWeb, the results of these models and detailed explanations of reference values are presented to the end-users as a complete set of information. If needed, end-users can exploit the detailed analysis of risk factors as in the total evaluation of the model (Figure 7 shows this analysis).

#### Combining Clinicogenomic Associations With the External Data

In ClinGenWeb, users can record and store additional types of risk factors (family history, environmental, behavioral, and clinical data) to assess the increase in their prostate cancer risk. When additional data is collected, it can be used as a parameter for the model or just included in the final report.

## Discussion

In this article of the miniseries, we have presented the design and development of the required structures for the NHIS-T to incorporate SNP and clinicogenomic data for disease risk assessment in the light of the first part of the miniseries, and of the analysis of the existing NHIS-T.

We have proposed the possible architectures and extensions of HL7 CDA templates to transmit personal, clinically relevant SNP data and clinicogenomic associations. In this approach, two important complementary capabilities are the structured knowledge base and the end-user assessment and reporting applications. The knowledge base (ClinGenKB) is responsible for transforming personal, clinically relevant SNP data to meaningful clinicogenomic associations. The end-user application (ClinGenWeb) ensures the issuing of personal reports where extracted clinicogenomic associations are listed, visualization of the risky SNPs, and the calculation of the total risk based on proposed risk models.

In the next part of this article, we will focus on the extraction of SNP associations to build the proposed ClinGenKB, and on the evaluation of the proposed components for the NHIS-T for determining prostate cancer risk using real direct-to-consumer SNP data files. In addition, assessment and reporting approaches to calculate personal prostate cancer risk will be presented.

## Multimedia Appendix 1

Cumulative Models for Prostate Cancer.

## Multimedia Appendix 2

Reference Tables for Cumulative Models.

## Multimedia Appendix 3

List of First Probabilistic Model Based Associations (Only SNP Model).

## Multimedia Appendix 4

List of Second Probabilistic Model Based Associations (SNP-Environmental Combined).

## Abbreviations

BioXM: BioXM Knowledge Management Environment
CCTS: Core Component Technical Specification
CDA: Clinical Document Architecture
ClinGenKB: Clinicogenomic Knowledge Base
ClinGenWeb: Clinicogenomic Web Application
CR-SNP: clinically relevant single nucleotide polymorphism
EHR: electronic health record
EMR: electronic medical record
HCRS: Health Coding Reference Server
HL7: Health Level 7
HL7 CDA R2: Health Level 7 Clinical Document Architecture release 2
ICD: International Classification of Diseases and Health Related Problems
MHDS: Minimum Health Dataset
MoH: Republic of Turkey Ministry of Health
NHDD: National Health Data Dictionary
NHIS-T: National Health Information System of Turkey
OR: odds ratio
PHR: personal health record
rsID: reference single nucleotide polymorphism identifier
SNP: single nucleotide polymorphism
TDS: Transmission Dataset
UN/CEFACT: United Nations Centre for Trade Facilitation and Electronic Business
V3: version 3
WES: whole exome sequencing
WGS: whole genome sequencing
XML: extensible markup language
